# Specialized Metabolites of the Lichen *Vulpicida pinastri* Act as Photoprotective Agents

**DOI:** 10.3390/molecules22071162

**Published:** 2017-07-12

**Authors:** Béatrice Legouin, Françoise Lohézic-Le Dévéhat, Solenn Ferron, Isabelle Rouaud, Pierre Le Pogam, Laurence Cornevin, Michel Bertrand, Joël Boustie

**Affiliations:** 1 ISCR-UMR CNRS 6226, Faculté des Sciences Pharmaceutiques et Biologiques, Institut des Sciences Chimiques de Rennes, Université Rennes 1, 2 Av. du Pr. Léon Bernard, 35043 Rennes CEDEX, France; solenn.ferron@univ-rennes1.fr (S.F.); isabelle.rouaud@univ-rennes1.fr (I.R.); pierre.lepogam.alluard@gmail.com (P.L.P.); laurence.cornevin@univ-rennes1.fr (L.C.); joel.boustie@univ-rennes1.fr (J.B.); 2 IETR-UMR CNRS 6164, Institut d’Électronique et de Télécommunications de Rennes, Université Rennes 1, 263 Av. du Général Leclerc, 35042 Rennes CEDEX, France; 3 French Lichenological Society, Station D’écologie Forestière, Route de la tour Dénecourt, 77300 Fontainebleau/Avon, France; bertrand.mic@wanadoo.fr

**Keywords:** lichen, photoprotection, antioxidant, phototoxicity, pulvinic acid, synergy

## Abstract

The extreme resiliency of lichens to UV radiations makes them an interesting model to find new photoprotective agents acting as UV-blockers and antioxidant. In this research, using a new in vitro method designed to overcome the shortage of material associated to many studies dealing with natural products, we show that the three major compounds isolated from the lichen *Vulpicida pinastri*, vulpinic acid, pinastric acid and usnic acid, were UV blocker agents. Antioxidant assays evidenced superoxide anion scavenging activity. Combination of the most promising compounds against UVB and UVB radiations, usnic acid, vulpinic acid and pinastric acid, increased the photoprotective activity. At the same time, they were found not cytotoxic on keratinocyte cell lines and photostable in the UVA and UVB ranges. Thus, lichens represent an attractive source to find good candidate ingredients as photoprotective agents. Additionally, the uncommon scalemic usnic acid mixture in this *Vulpicida* species was proven through electronic circular dichroism calculation.

## 1. Introduction

Since the birth of Life on Earth, Ultraviolet radiations (UV) threatened the fragile equilibrium between a physiological need of UV and their harmful effects on DNA acting by a direct energetic effect or by generation of reactive oxygen species [1]. Incidence rates of melanoma, one of the most deleterious skin cancer on humans, are projected to rise caused by acute and chronic UV exposure [2]. Among the preventive strategies, UV blockers remain commonly used. These compounds are expected to act like filters covering the broad UVA–UVB range and to present antioxidant properties. They must also be photostable without any dermal toxicity. Currently, a sunscreen is only guaranteed by a combination of different organic and mineral filters, these latter being essential to reach a high index of protection. Many concerns about UV filters safety exist [3,4] and their role in damaging aquatic life [5,6]. Thus, it is mandatory to find new molecules responding to a growing consumer demand focused on natural sources. Some natural ingredients [7], mainly issued from plants [8] or algae [9], can already be found in commercialized sunscreens [6,10]. Indeed, among strategies implemented by animals and plants to counteract the deleterious effects of UV radiations, the production of mineral physical UV blockers, e.g., calcium oxalate crystals, or organic UV absorbing metabolites can be observed [11]. Some of them could additionally neutralize the radical oxygen species Thus, flavonoids can be cited for plants, mycosporines or eumelanins for animals and fungi [12]. As pioneer species in extreme places, with high light-irradiance and thus elevated UV exposure, lichens are of interest to this field. Lichen compounds can be a good source of inspiration for potent UV blockers for organic chemists and biotechnological options can be envisaged in some cases [13]. However, a possible shortage of this low growing resource has to be considered.

The extreme resiliency of lichens is partly based on the biosynthesis of various unique compounds, comprising a wide array of photoprotective metabolites. Owing to the presence of both pulvinic acid derivatives suspected to protect from UVA and usnic acid known to be a UVB blocker, we selected for investigation the lichen *Vulpicida pinastri* (Scop.) Mattson & Lai, a sun exposed yellow lichen growing at high elevations in mountain forests [14]. Direct Analysis in Real Time (DART-HRMS) [15,16] analyses carried out on unprocessed pieces of thallus confirmed the presence of vulpinic (**1**) and pinastric (**2**) acids, two pulvinic acid derivatives, and usnic (**3**) acid within the sample studied herein, paving the way for their subsequent isolation. The three major compounds, both alone and in combination, were then tested in vitro for their photoprotective activities. Whereas in vivo Sun Protection Factor (SPF) is considered as the standard, alternative in vitro methods, based on photoprotective indexes calculations (Sun Protection Factor (SPF), PF-UVA, and critical wavelength (λc)), are recognized by cosmetic organizations [17,18,19]. A homemade in vitro method was preferred to determine the photoprotection indexes for technical reasons and minute material amount. Antioxidant capacities were evaluated through 1,1′-diphenyl-2-picrylhydrazyl (DPPH) test and scavenging activity on superoxide anion. Then, to assess their possible use in cosmetics, compounds **1**–**3** were evaluated through the Photo-Irritancy Factor (PIF) for their safety on human keratinocytes before and after UVA irradiation as well as for their photostability. As preliminary results, the study reveals UVA booster properties for pulvinic acids while usnic acid was confirmed as UVB blocker and highlights synergistic effect when compounds were tested in combination. Finally, products exhibit antioxidant activities without cytophototoxicity and their photostability was attested. Lichens are evidenced as a promising source of photoprotective compounds.

## 2. Results and discussion

### 2.1. Detection and Isolation of the Major Lichen Compounds

**Figure 1:**
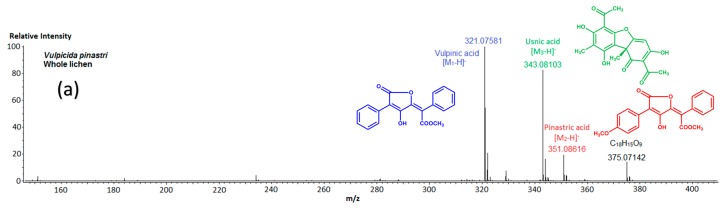

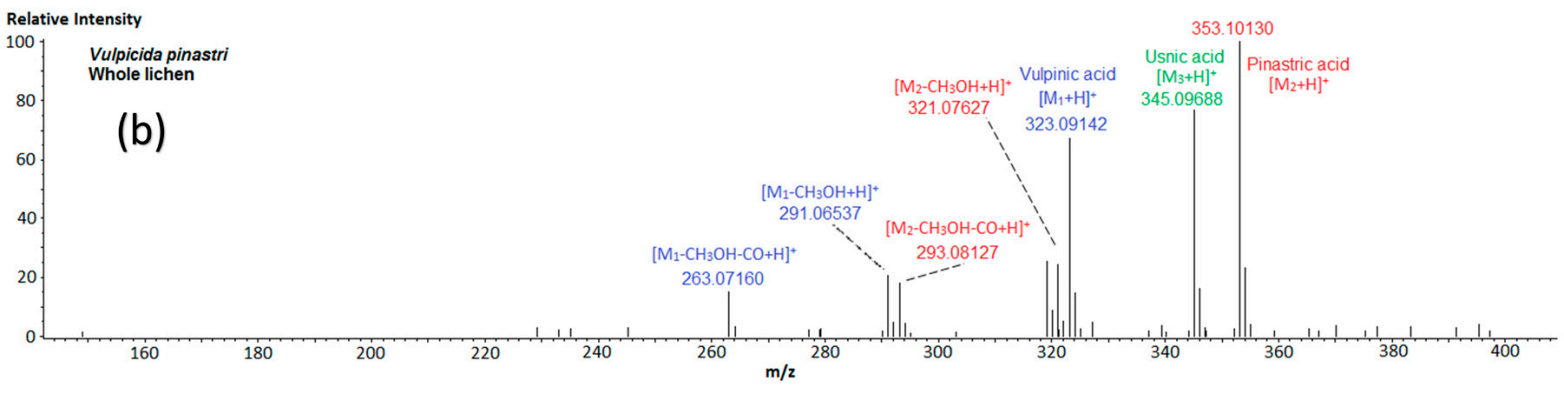
DART mass spectra (*m*/*z* 150–400) of the thalli of *Vulpicida pinastri* in: (**a**) negative ionization mode; and (**b**) positive ionization mode.

DART-HRMS analyses undertaken from complete pieces of *Vulpicida pinastri* in both positive (PI) and negative (NI) modes enabled the straightforward detection and identification of its main secondary metabolites (Figure 1).

The values of *m*/*z* ratios of the major peaks detected in negative ionization mode are in agreement with the formation of the [M − H]^−^ ions from three lichen compounds formulae vulpinic acid **1** and pinastric acid **2**, two pulvinic acid derivatives and usnic acid **3**, related to dibenzofuran derivatives [20] (Table 1).

Likewise, PI-DART-MS exhibited prevalent protonated molecules for all these metabolites but some peaks of notable intensity corresponding to putative fragments arose as well (Figure 1 and Table S1 in Supplementary Materials (SM)). Among these, the signals at *m*/*z* 291 and 321 are indicative of the formation of dilactone derivatives through the loss of methanol from vulpinic and pinastric acids as respective parent compounds, consistently with previous reports on closely related metabolites [21,22]. Subsequent neutral losses of CO from these latter fragments, respectively, account for *m*/*z* 263 and 291, as described elsewhere [23].

The major compounds being evidenced in the sample, the extraction was conducted as described in Section 3.3 on 5.2 g of the powdered lichen and provided a crude extract with a yield of 6.7% which is a significant proportion comparatively to usual extraction yields [24]. The three main compounds, **1**, **2** and **3**, were separated through successive chromatographies and identified by NMR.

NMR spectra (See spectra S1–S3 and Table S2 in SM) enabled a distinction to be made between compounds **1** and **2** that differ one from another by the presence of a methyl group OCH_3_-4 for **2** (δ = 3.85 ppm) [25,26].

**Figure 2:**
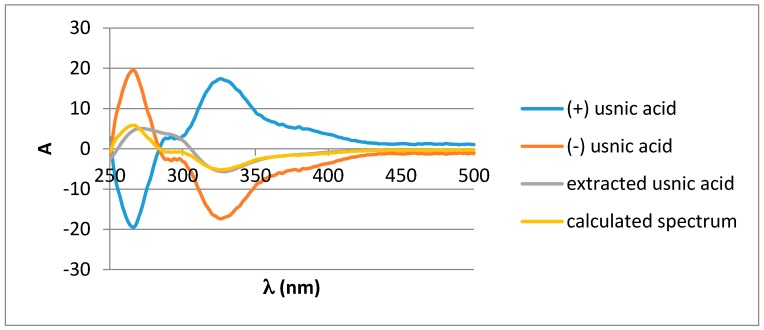
Electronic circular dichroism in CHCl_3_ at 39 µM. The theoretical spectrum was calculated considering a 35:65 ratio for (+)/(−) usnic acid.

Identification of usnic acid by NMR (spectrum S4 in SM) and HPTLC resulted identical to an authentic commercial standard [27] except for the optical rotation of usnic acid in chloroform at 20 °C. The experimental value was [α]${}_{D}^{25}$ = −87.6 mL·dm^−1^·g^−1^ while the expected value at 25 °C was [α]${}_{D}^{25}$ = ±495.0 mL·dm^−1^·g^−1^ for the pure dextrogyre or levogyre enantiomer. This value suggested a mixture of both (+) and (−) isomers in a ratio 40:60. Comparing the experimental electronic circular dichroism spectrum and the calculated one confirms the existence of both (+) and (−) conformers in *V. pinastri*, in a 35/65 ratio (Figure 2) [28,29].

### 2.2. Lichen Compounds and Extract as UV-Blockers

**Figure 3:**
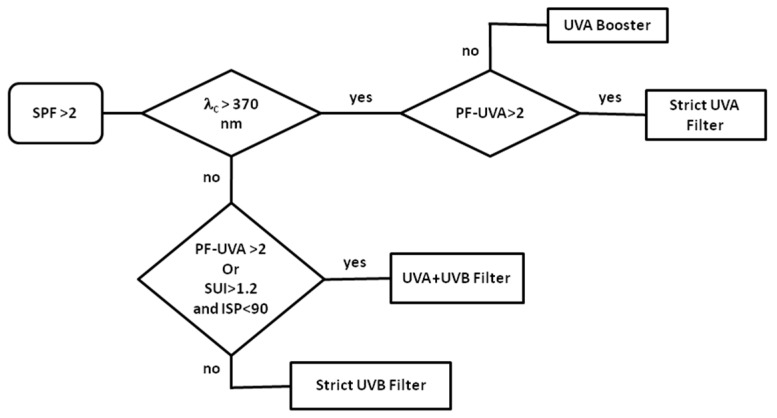
Decision tree for predicting the UV protection range of a compound from its photoprotection indexes.

Photoprotective effectiveness is evaluated through photoprotective indexes such as SPF, PF-UVA, and critical wavelength (λ_c_) (an indicator of the filter capacity to protect in the UVA range), as well as Spectral Uniformity Index (SUI) and Ideal Spectral Profile (ISP) [30,31], two indexes recently defined by Diffey to focus on protection across the entire ultraviolet spectrum. They calculate the goodness of fit of the spectral profile to the ideal flat profile. SPF and PF-UVA measure the filter efficacy against UVB or UVA. SPF measurement is currently determined in vivo through determination of the erythemal reaction on human volunteers. For economical and ethical reasons, many alternative in vitro methods have been developed to measure SPF and also PF-UVA, λc or SUI and ISP. Unfortunately, despite concerted efforts around the globe between different commissions, none of them guarantees a good correlation with in vivo measurements [32]. Moreover, all these in vitro methods require an amount of active raw material generally ≥100 mg and prevent the evaluation of compounds in minute amounts. The photoprotective indexes have been then determined thanks to a new method (see SM) especially developed to cope with the low availability of the compounds. Twelve commercial organic UV-filters, the photoprotective indexes of which were published [33,34] (Table S3 in SM), encompassing a 1–24 SPF range were used for the development. It can be noted that the SPF values of benzophenone-3 (oxybenzone), PEG-25 PABA, homosalate, polysilicone 15 and 4-Methylbenzylidene Camphor are under 6, the value commonly accepted as the lower limit for commercial sunscreen. However, the efficient value of commercialized emulsion can be explained by the formulation of several organic and mineral filters. The similarity of the results (r^2^ = 0.98 for published SPF vs. herein SPF, see Figure S1 in SM) supported the new method validity. Routinely, ethylhexyl methoxycinnamate (OMC) was used as positive control. Based on the SPF, λc, PF-UVA, SUI and ISP values obtained with marketed organic filters and on their UV range protection (Table S3 in SM), a decision tree was built to categorize the filters (Figure 3). Considering the herein SPF values of the filters cited above (around 2), the first condition to select a candidate is SPF > 2. Restricted UVA or UVB filters can be discriminated through the λc value (>370 nm) and PF-UVA (>2) for UVA and SUI (>1.2) and ISP (<90) for UVB. A large spectral protection (UVA + UVB) could be evaluated by combining the four criteria.

**Figure 4:**
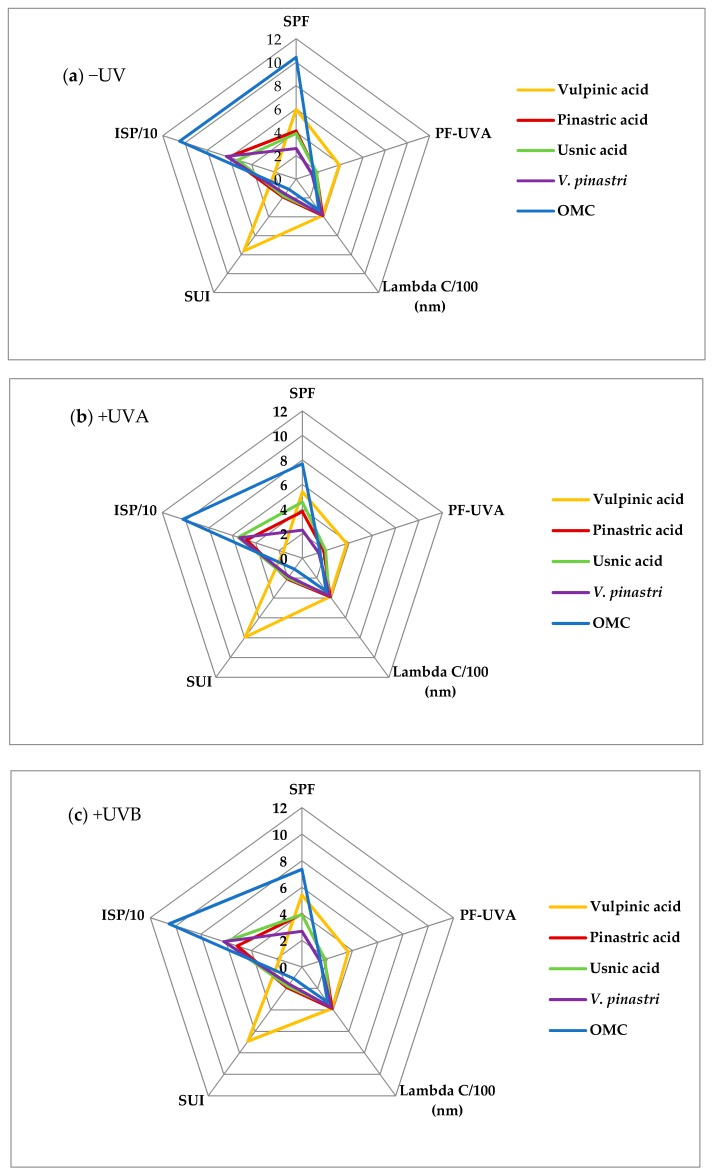
Photoprotection index profiles: (**a**) before; and (**b**) after UVA; and (**c**) after UVB irradiation of *Vulpicida pinastri* extract and its three major compounds. (For scale reason, the critical wavelength values and the ISP values are divided by 100 and 10 respectively).

Regarding the photoprotective index profiles (Figure 4), a first outcome is the striking difference of vulpinic acid profile. Its high SUI value (7.6), low ISP value (19%) and high PF-UVA value (3.9) are indicative of a UVA filter. This assessment is in agreement with the outcome of the decision tree (Figure 3). Surprisingly, the SPF and PF-UVA values of vulpinic acid are higher than the values found previously [35]. In this case, these values had been determined thanks to an in vitro method in which the compound was included into an O/W emulsion. An undetected solubility problem can be suggested given that vulpinic acid is an apolar compound. The poor solubility of lichen metabolites is a recurrent pitfall to assess their bioactivities [36] and Rancan et al. experimented in two different solvents to face the low solubility of products [37].

The photoprotective index profiles of usnic acid, pinastric acid and OMC are similar (Figure 4a). The decision tree led us to classify pinastric acid (SPF = 4.1, λc = 386 nm, PF-UVA = 1.8, ISP = 61% and SUI = 1.9) as a UVA booster due to its high critical wavelength and its low PF-UVA while usnic acid (SPF = 3.9, λc = 364 nm, PF-UVA = 1.8, ISP = 53% and SUI = 1.8) is categorized as a UVB filter like the positive control OMC (SPF = 10.4, λc = 330 nm, PF-UVA = 1.6, ISP = 105% and SUI = 1.1). These results corroborate the photoprotective properties of the pulvinic acid derivatives, calycin being described as a promising UVA filter [37,38]. Rancan found an in vivo SPF value of 4.1 in good agreement with our value of 3.9 for usnic acid which also supports the new method we use to estimate photoprotection indexes. The *Vulpicida pinastri* extract (SPF = 2.6, λc = 378 nm, PF-UVA = 1.5, ISP = 63% and SUI = 1.6) contains pinastric acid as major compound and exhibits the same UVA booster property as this pulvinic acid derivative.

### 2.3. Photostability of the Compounds

Photostability is an important feature to develop sunscreens, for example OMC and butyl methoxydibenzoylmethane (avobenzone) are known to lose their photoprotective properties when irradiated by UV [39]. Thus the compounds photostability was evaluated through photoprotection indicators calculation after UVA or UVB irradiation. As expected (Figure 4b,c), OMC exhibits a 25% decrease in indexes after irradiation which demonstrates its photoinstability attributed to its *cis*/*trans* photoisomerization [40]. The SPF values of both pulvinic acid derivatives **1** and **2** present the same little SPF variation. It can be related to the slight deformation of their spectra in the UVB region after UVA or UVB irradiation (see Figure S4 in SM). Regarding the usnic acid spectra (see Figure S4 in SM), a different shape was observed in the 290–400 nm range after UVA irradiation evidencing a likely photodegradation product. This is consistent with Fernandez who reported the formation of an usnic acid’s photoproduct after irradiation [41]. This could explain the +17% variation in the SPF values after UVA irradiation. As *Vulpicida pinastri* extract contains usnic acid, a 12% decrease of its SPF value after UVA irradiation was observed due to its higher contents in pulvinic acid derivatives. Nevertheless, all compounds retain their photoprotective properties compared with OMC which could also contribute to the resilience of this lichen under sunny conditions at high altitude [14].

### 2.4. Antioxidant and Phototoxic Activities of Lichen Compounds and Extract

Specialized metabolites with antioxidant activity are known to play a role in photoprotection as UV-damages are partly mediated by reactive oxygen species [42]. Two acellular and colorimetric methods were used to evaluate this capacity, reducing power through DPPH assay and superoxide anion scavenging through NBT test on the isolated compounds **1**–**3** and the *Vulpicida pinastri* extract (Table 2). Vulpinic acid and pinastric acid, to a lesser extent, appeared to be moderate antioxidant compounds on DPPH test compared with the positive control ascorbic acid (IC_50_ = 55.0; IC_50_ = 80.0 vs. IC_50_ = 12.5 µg/mL, respectively). Nevertheless, this activity was better or equal to *Vulpicida pinastri* extract while usnic acid is confirmed to have no activity on DPPH (inactive below 500 µg/mL) [43]. Interestingly, the crude extract exhibited an overall antioxidant activity of the same magnitude than that of the isolated compounds suggesting a synergistic photoprotective effect. Towards the superoxide anion, a better scavenging activity was recorded for all compounds (IC_50_ = 30; IC_50_ = 70; IC_50_ = 24 µg/mL for vulpinic, pinastric and usnic acids, respectively) but lower than the reference compound ascorbic acid (IC_50_ = 3 µg·mL^−1^). This antioxidant activity is described for the first time for these two pulvinic acid derivatives and confirmed the antioxidant studies focused on norbadione [44,45,46,47,48].

As a preliminary indication for safety, phototoxicity of the compounds **1**–**3** and of the *Vulpicida pinastri* extract was evaluated through the calculation of the photo-irritancy factor (PIF) on a human keratinocyte cell line before and after UVA irradiation. Except for the extract for which a >PIF is calculated indicating a phototoxic potential, all metabolites were under the threshold value of 5. The pulvinic acid derivatives and usnic acid had no phototoxic potential. These results are in agreement with Varol [49] and Mitchell [41,50].

### 2.5. Synergistic Activity of Lichen Compounds and Extract

An effective photoprotection is generally guaranteed by a large UV range cover thanks to a series of efficient filters. Considering the UV profile of the three major metabolites, a mixture with a restricted UVB filter (usnic acid) and restricted UVA filter (vulpinic acid) appears to cover the 290–400 nm range for which the synergistic combination is here studied. The results obtained with the mixture usnic acid–pinastric acid (which exhibited the same UV profile than vulpinic acid) are presented in SM and are reminiscent of those of vulpinic–usnic acids mixture (Figure S5 and S6 in SM).

#### 2.5.1. UV-Blockers

The UV-blocker properties are appreciated through photoprotective indexes for which we compared experimental results with calculated values obtained through the method described (see SM).

**Figure 5:**
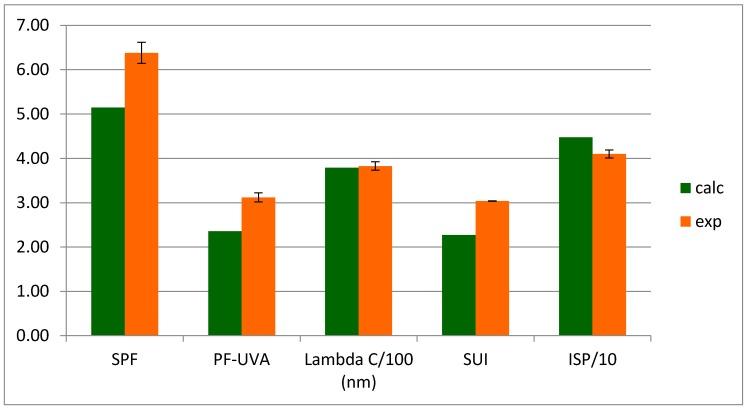
Calculated and experimental photoprotective indexes of the vulpinic and usnic acids mixture (**1** + **3**) (cal: calculated; exp: experimental).

**Figure 6:**
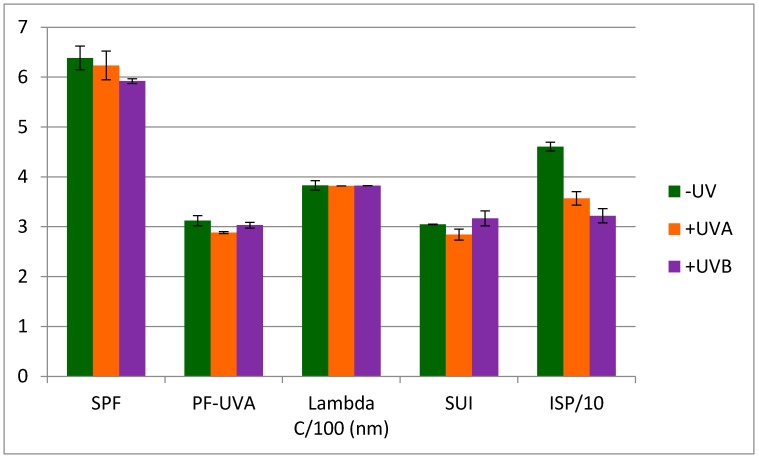
Experimental photoprotection indexes of a mixture of vulpinic and usnic acids (**1** + **3**) before (−UV) and after UVA or UVB irradiation (*n* = 2).

Except ISP values, all experimental indexes exhibited a higher value than the calculated ones (Figure 5). SPF indexes are more than 30% higher while PF-UVA values are more than 20% higher which is indicative of a real synergy. The enhancement is observed for all indexes; before and after UV irradiation, the index values remained constant upon irradiation (Figure 6). Such a synergistic interaction is described for the first time for lichen compounds while it has been already described for compounds issued from the vegetal realm [51,52].

#### 2.5.2. Antioxidant Activity

The UVB blocker usnic acid and the most potent anti-UVA compound, vulpinic acid, were selected in order to determine the extent of synergism for superoxide anion scavenging according to a combination effect in different ratios (Table S5 in SM). Using the isobologram analysis method, we determined the combination index values (CI) for these different ratios.

**Figure 7:**
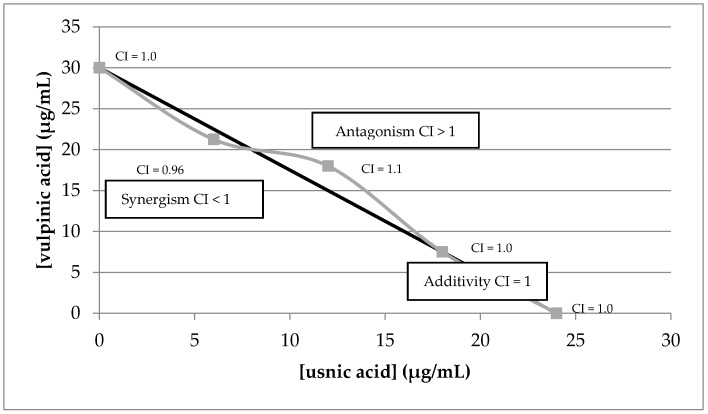
Isobologram analysis to evaluate the combination effect of vulpinic acid and usnic acid on superoxide scavenging activity (evaluated via NBT assay).

Figure 7 shows that these two compounds were mostly additive in a ratio of usnic acid and vulpinic acid of 1:0.4 but a moderate antagonism could be observed in a ratio 1:1.5. Thus, it can be concluded that there was 2–4-fold lower concentration requirement in combination ratio between usnic acid and vulpinic in order to show a synergistic effect.

Combinations of pinastric acid and usnic acid were also evaluated (Figure S7 and Table S6 in SM) and a clear synergistic effect was observed in a ratio of usnic acid and pinastric acid of 1:7.5 to 1:1.5. Thus, it can be concluded that there was 1–8-fold lower concentration requirement in combination ratio between usnic acid and vulpinic acid in order to show a synergistic effect but the optimum of synergy was observed with a ratio of 1.5.

#### 2.5.3. Photocytotoxicity of Usnic Acid and Pinastric Acid

**Figure 8:**
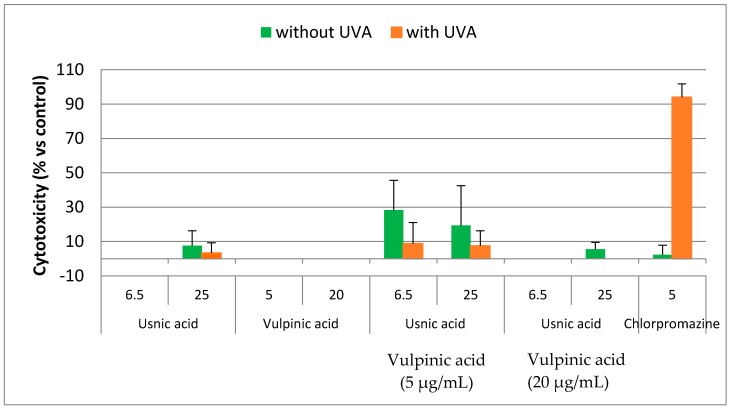
Cytotoxicity on HaCat cell line of vulpinic acid (**1**) and usnic acid (**3**) and in combination before and after irradiation under UVA (*n* = 3).

The combination of usnic acid with respectively vulpinic acid and pinastric acid were evaluated before and after UVA irradiation at 25%, 50%, 75% and 100% of the IC_50_ on HaCaT keratinocytes cell line. We reported here the results obtained for the synergistic combination between vulpinic acid and usnic acid in the range of active antioxidant concentration (Figure 8). Before irradiation, the cytotoxicity observed after combination of both compounds increased slightly without reaching the IC_50_ (Table S7 in SM). After irradiation by UVA, the cytotoxic activity decreased compared with the positive control chlorpromazine. Similar results were obtained with pinastric and usnic acids (Table S8 and Figure S8 in SM). Thus, lichen compounds were not cytotoxic even in combination.

## 3. Materials and Methods

### 3.1. General

The solvents required for lichen extraction (acetone, dichloromethane, chloroform) were of analytical grade as so as absolute ethanol, dimethyl sulfoxide (DMSO), tetrahydrofuran (THF), sodium dodecyl sulfate (SDS) purchased from Sigma (St Quentin-Fallavier, France) and paraffinum liquidum obtained from Cooper (Melun, France). To prepare the O/W emulsion, double-distilled water was used. All commercial UV filter origins were reported in Table S3 (See SM). (+)-usnic acid was purchased from Sigma (St Quentin-Fallavier, France) and (−)-usnic acid was obtained from the laboratory’s chemical library and referenced as JB/A/007.

Chromatographic separations were performed using vacuum liquid chromatography on silica gel (Merck 35–70 μm). The preparative chromatography was coated with silica (Silica GF_254_, 35–70 µm, Merck 7730) to a thickness of 3 mm. Analytical TLC plates (Merck silica gel 60F254) were eluted using standard solvent systems (CHCl_3_/acetone: 4/1 and *n*-Hexane/Ethylether/formic acid: 130/80/20) [53,54]. Compounds were evidenced under UV light (254 and 365 nm) and/or after spraying anisaldehyde-H_2_SO_4_ reagent prior to heating. Melting points were measured on a Kofler hot-stage apparatus. Optical rotation was determined with a Perkin-Elmer model 341 polarimeter. UV spectra were recorded on a Uvikon 931 spectrophotometer or a SpectroStar nano microplate reader. UVA and UVB irradiation were performed with a stratagene UV Stratalinker 2400 crosslinker. Electronic circular dichroism spectra were recorded using a Jasco J-815 ECD spectrometer. ^1^H- and ^13^C-NMR spectra were recorded at 500 and 125 MHz on a Bruker DMX 500 WB NMR spectrometer, at 400 and 100 MHz on a Bruker Avance III 400 or at 300 and 75 MHz, on a Bruker 300 NMR spectrometer respectively, using CDCl_3_, DMSO-*d*_6_, acetone-*d*_6_ or methanol-*d*_4_ as solvents. Mass spectra analyses were performed using a JEOL JMS-T100CS (AccuTOF CS) orthogonal time-of-flight (TOF) mass spectrometer (Peabody, MA, USA) equipped with an IonSense DART Source (Danvers, MA, USA). Ultra-high purity helium was used as reagent gas at a flow rate of 4 L·min^−1^ and under a temperature value of 523 K. The following DART-needle, discharge electrode, and the grid electrode voltage values were used: 3500, 150 and 250 V, respectively. The voltage values of orifice 1, orifice 2, and the ring lens were set at 15, 5 and 10 V, respectively. The orifice 1 temperature was kept at 353 K. The detector voltage was set at 2300 V. The mass spectra were recorded every second with a resolution of 6000 fwhm. The mass scale was calibrated using the [M + H]^+^ ion series of a poly(ethylene glycol) diluted in a CH_2_Cl_2_/MeOH mixture (1:1) and [M − H]^−^ ion series of a poly(ethyleneglycol) sulfate in negative-ion mode. To perform accurate mass measurements, the mass drift compensation procedure available on the main program that controls the AccuTOF CS was used to compensate for the *m*/*z* drift in the range of *m*/*z* 100 to 500. DART-MS analyses of the intact pieces of *V. pinastri* were performed by holding unprocessed pieces of lichen between tweezers directly under the helium stream.

### 3.2. Lichen Material

*Vulpicida pinastri* (Scop.) Mattson & Lai was collected and identified by Michel Bertrand the 27/04/2014 near the Lac des Sagnes (Jausier, Alpes de Haute Provence, France), on a larch bark. The voucher specimen was stored under dried conditions in the dark and referenced in the laboratory under number JB/14/198.

### 3.3. Vulpicida pinastri Extraction and Isolation of Its Major Compounds ***1***, ***2***, ***3***

The air-dried whole thalli of *Vulpicida pinastri* (5.2 g) were packed in a filter paper cartridge in a Soxhlet apparatus and extracted at reflux with acetone/dichloromethane (1/1) during three hours (2 times × 200 mL). Both extracts were mixed and evaporated in vacuo (residue = 348 mg). One part (100 mg) was chromatographed on a vacuum liquid chromatography silica gel column (150 g, 2 × 20 cm) and eluted by diethyl ether-chloroform (100:0 to 2:98) as the mobile phase. Four fractions (VP1-VP4) were obtained. Fraction VP2 (22 mg) was selected for further chromatography through preparative TLC using chloroform/acetone (3/1) and yielded vulpinic acid (**1**, 1.6 mg) and pinastric acid (**2**, 2.6 mg). Fraction VP4 afforded usnic acid (**3**, 16.0 mg).

### 3.4. Electronic Circular Dichroism

Electronic circular dichroism spectra were recorded in CHCl_3_. (+)- and (−)-usnic acid solutions (39.2 mM) were used as references and allowed the construction of a theoretical spectrum to fit the experimental one. The experimental spectrum was recorded from usnic acid extracted from *V. pinastri* (39.2 mM).

### 3.5. In Vitro Sun Protection Indexes

#### 3.5.1. In Vitro Screening Method for Sun Protection Indexes Evaluation

A new in vitro screening method based on spectrophotometric data was used to evaluate sun protection indexes such as SPF, PF-UVA, the critical wavelength λc, ISP and SUI. Its development was first conducted in a cuvette using twelve commercial organic filters encompassing a large range of SPF values (1 < SPF < 25) and then transposed in a 96-wells plate. Following the International recommendations [55], maximal concentrations never overtook 10% whatever the commercial filters or the lichens extracts or compounds considered. This new method faces some product availability limitation, which is a common shortcoming for natural products or metabolites (see all development in SM).

**Figure 9:**
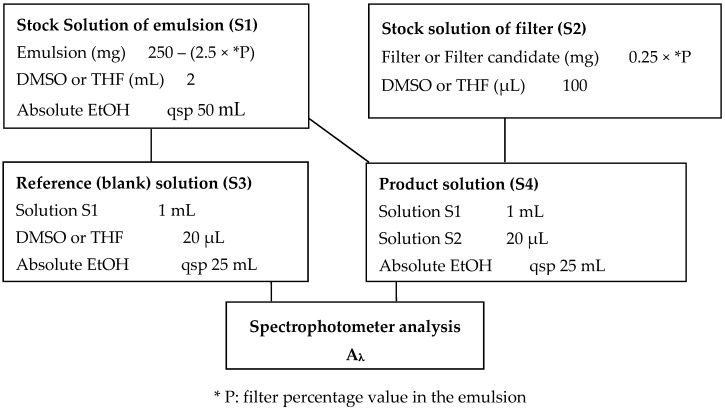
Preparation of sample solution for experimental UV absorbance measurements.

The scheme of solution preparation to record the experimental values is depicted in Figure 9. The sample (S4) was prepared at 0.02 mg/mL from stock solutions of filter candidate (S2) and of emulsion (S1) (See emulsion preparation in SM) taking into account the maximal concentration. The experimental absorbances A_λ_ were recorded in the 290–400 nm range versus a blank solution (S3) in a cuvette with a double beam spectrophotometer or in a 96-wells plate filled with 180 µL of solution by well. One can note that the samples were first dissolved either in THF (*V. pinastri* extract and usnic acid) or in DMSO (OMC, pinastric acid and vulpinic acid) for solubility reason (S2). The diagram is described for final volumes of 50 mL, 100 µL, 25 mL and 25 mL for S1, S2, S3 and S4 solutions, respectively. Smaller volumes can easily be prepared. Solutions S2 and S4 were prepared in duplicate. In case of lichen compounds and extract, OMC was used as positive control.

The experimental data (A_λ_) were transferred into a dedicated Excel spreadsheet to get photoprotection indexes. The experimental values have directly resulted in both SPF and PF-UVA value, critical wavelength, Spectral Uniformity Index, Ideal Spectral Profile (See SM).

#### 3.5.2. Stability of the Tested Compound after UVA or UVB Irradiation

One hundred eighty microliters of solution in 96-well plates were irradiated by UVA (2 J/cm^2^) or UVB (100 mJ/cm). The absorbances were then recorded and photoprotective indexes were calculated as described above. The stability was evaluated by comparison between indexes described previously (Section 3.5.1) and obtained before and after irradiation. Two independently prepared solutions were studied and OMC was used as a positive control.

#### 3.5.3. Synergistic Study

Experimental and theoretical combinations between usnic and pinastric acids on one hand and usnic and vulpinic acids on the other hand were considered. Five percent of each compound were combined in solution to reach a maximum of 10% in the well. The absorbances were recorded to lead to experimental indexes after mathematical treatment. Theoretical absorbances of the 10% compounds mixtures were calculated by summing experimental values obtained from each molecule tested alone at 5%. These calculated data were then transferred into the Excel spreadsheet to get the expected indexes of the mixture. The synergism was evaluated by comparing these results with the experimental ones. The evaluation was conducted before and after UVA and UVB irradiation. Each determination was done in duplicate and averaged.

### 3.6. Antioxidant Testing

#### 3.6.1. Single Compounds

Reducing activity of compounds **1**–**3** and crude extract on the 1,1′-diphenyl-2-picrylhydrazyl free radical (DPPH) and scavenging activity on superoxide anion were measured as previously described [35]. Briefly, the scavenging activity of the lichen compounds on DPPH was measured using the Matsukawa method [56] with some modifications [57]. A reaction mixture containing 100 µL of DPPH (0.5 mM) in methanol and 10 µL of the lichen compound solutions in DMSO to give final concentrations of 19, 56, 167 and 500 µg/mL per well, was distributed in each microplate well. To allow results comparison and due to the presence of extract, concentrations are expressed in µg/mL. The absorbances were recorded at 540 nm after 30 min.

Measurements of superoxide anion scavenging activity in 96-well microplates was based on the non-enzymatic method previously described with some modifications [57,58]. The reaction mixture in the sample wells consisted of NADH (78 µM), nitro-blue tetrazolium (NBT) (50 µM), phenazine methosulfate (PMS) (10 µM), and lichen compounds (10, 20, 40, 80 µg/mL). The reagents were dissolved in 16 mM tris-hydrochloride buffer, at pH = 8 except for all the lichen compounds/extract which were dissolved in DMSO. After 5 min of incubation at room temperature, the spectrophotometric measurement was performed at 560 nm against blanks (without PMS nor sample).

For both assays, each concentration and all tests were done in triplicate and the results averaged. Ascorbic acid was used as a positive control. The percentage inhibition at steady state for each dilution was used to calculate the IC_50_ values. This gave the amount of antioxidant required (measured as the concentration of the stock solution added to the reaction mixture) to either scavenge 50% of O_2_^−^ or 50% of DPPH radicals: the lower the values, the higher the scavenging activity towards O_2_^−^.

#### 3.6.2. Compounds in Combination

The effect of combination between each pulvinic acid derivative (**1**,**2**) with usnic acid (**3**) was determined at 25%, 50%, 75% and 100% of their IC_50_ for NBT antioxidant assay. For each drug ratio (Tables S5 and S6 in SM), the antioxidant inhibition was evaluated as previously described (2.6.1). An isobologram was constructed graphically: doses of compounds A and B gave abscissa and ordinate respectively and the effect of drug combinations is plotted as graph. The experimental curve was compared to the line of IC_50_ representing the line of additivity (25% of IC_50_ (**1**) + 75% of IC_50_ (**2**) = 100 of IC_50_ (**1**,**2**)) and built by construction. 

The antagonistic, synergistic or additive effect of the combination depends on the combination index value (CI) (Equation (1)) [59]. A CI = 1 indicates that the effect of one drug is additive to the second, antagonism between two agents is defined by a CI > 1 while a CI < 1 means synergism between the agents [60].

(1) $$\mathrm{CI}=\frac{D\left(A\right)}{{\left({\mathrm{IC}}_{50}\right)}_{A}}+\frac{D\left(B\right)}{{\left({\mathrm{IC}}_{50}\right)}_{B}}$$

D(A) and D(B) are the doses of compound A and B, respectively that produce 50% inhibition when given together. (IC_50_)_X_ is the value of the IC_50_ of compound X alone.

For both assays, each concentration and all tests were done in triplicate and the results averaged.

### 3.7. Phototoxicity

#### 3.7.1. Single Compounds

Cytotoxic and photocytotoxic activities of *Vulpicida pinastri* extract and of compounds **1**–**3** were evaluated according to the OECD guideline [61] as previously described [35]. Briefly, 100 µL per well of a cell suspension of HaCaT cells (ATCC) at 120,000 cells/mL were maintained in a RPMI culture medium with 5% of calf serum at 37 °C under 5% CO_2_ for 24 h for formation of monolayers. At t = 24 h, two 96-well plates per test chemical were preincubated for 1 h with seven different concentrations of the tested compounds and extract (ranging from 1 µg/mL to 200 µg/mL). Chlorpromazine was used as the positive control. To allow results comparison and due to the presence of extract, concentrations are expressed in µg/mL. To predict the phototoxic potential, we determined the Photo-Irritancy Factor (PIF) thanks to the concentration response curves obtained in the presence and in the absence of radiation. If PIF < 5, no phototoxic potential is predicted. If both IC_50_ (−UV) and (+UV) cannot be calculated, a formal PIF (PIF = *1) is used predicting no phototoxic potential. If a chemical is only toxic under UV but exhibited no cytotoxicity without UV, the PIF cannot be calculate and a >PIF is calculated using the highest concentration. A phytotoxic potential is so suggested.

For both assays, each concentration and all tests were done in triplicate and the results averaged. The percentage inhibition at steady state for each dilution was used to calculate the IC_50_ values.

#### 3.7.2. Compounds in Combination

The effect of combination between each pulvinic acid derivative (**1**,**2**) with usnic acid (**3**) was determined at 25%, 50%, 75% and 100% of their IC_50_ for cytotoxicity assay without irradiation. For each drug ratio (Tables S7 and S8 in SM), the cytotoxicity was evaluated as previously described (2.7.1).

For both assays, each concentration was done in triplicate and the results averaged.

## 4. Conclusions

In summary, the lichen *Vulpicida pinastri* contains secondary metabolites that can block UVA and UVB but are also additive or synergistic as antioxidant. The lichen model appears to be promising to find new photoprotective compounds. The three major compounds usnic, vulpinic and pinastric acids studied here are photostable and not photocytotoxic. (i) The scalemic mixture of usnic acid is described for the first time in *Vulpicida pinastri*; and (ii) the photoprotection indexes calculation strategy described herein might be beneficial in the wider realm of natural products, the paucity of which often precludes the assessment of their biological activities.

## Figures and Tables

**Table 1:** Results of exact mass measurements performed from the mass spectrum of the Figure 1 related to the NI-DART-MS of a whole piece of *Vulpicida pinastri*. Note that the detected species arose as singly charged species.

Compound	Measured Mass	Proposed Formula	Calculated Mass (Error in ppm)
**Vulpinic acid**	321.07581	C_19_H_13_O_5_	321.07630 (−1.53)
**Pinastric acid**	351.08616	C_20_H_15_O_6_	351.08686 (−2.01)
**Usnic acid**	343.08103	C_18_H_15_O_7_	343.08233 (−3.24)

**Table 2:** Antioxidant, cytotoxic and phototoxic activities of *Vulpicida pinastri* extract and its three major compounds.

Compound	Antioxidant Activities		Phototoxic Activities on HaCaT Cells		
	DPPH Assay IC_50_ ± SD (µg/mL)	NBT Assay IC_50_ ± SD (µg/mL)	IC_50_ ± SD (µg/mL)		Photo-Irritancy Factor (PIF)
			Without Irradiation	With Irradiation	
**Vulpinic acid**	55.0 ± 8.0	30.0 ± 3.0	>200.0	>200.0	*1.0 ^c^
**Pinastric acid**	80.0 ± 8.0	70.0±23.0	>200.0	>200.0	*1.0 ^c^
**Usnic acid**	>500.0	24.0 ± 9	33.0 ± 2.0	47.0 ± 10.0	0.7 ^d^
***V. pinastri* extract**	75.6 ± 0.6	65.0 ± 5.0	>200.0	58.0 ± 2.0	>3.4 ^e^
Ascorbic acid ^a^	12.5 ± 0.5	3.0 ± 0.4	-	-	-
Chlorpromazine ^b^	-	-	13.2 ± 1.7	2.4 ± 0.3	5.5 ^d^

^a^ Antioxidant positive control; ^b^ Cytotoxic positive control; ^c^ formal PIF = *1.0 = C_max_(−UV)/C_max_(+UV); ^d^ PIF = IC_50_(−UV)/IC_50_(+UV); ^e^ >PIF = C_max_(−UV)/ IC_50_(+UV).

## Supplementary Materials

The following are available online. PI-DART-MS data of *Vulpicida pinastri* are given in Table S1; NMR spectra and data are given in Spectrum A1 to A4 and Table S2; Figures S1 to S4 and Tables S3 and S4 are related to the validation of the new in vitro method; Figures S5 to S8 and Tables S5 to S8 correspond to the study of lichen compounds in combination.
