# Supplementary material for: Specialized Metabolites of the Lichen Vulpicida pinastri Act as Photoprotective Agents
Source: Molecules. 2017 Jul 12;22(7):1162. doi: 10.3390/molecules22071162 (PMC6152234; doi:10.3390/molecules22071162)
Supplement: Supplementary file 1 [file molecules-22-01162-s001.pdf]

molecules

Specialized Metabolites of the Lichen *Vulpicida pinastri* Act as Photoprotective Agents

Béatrice Legouin [1,*,†], Françoise Lohézic-Le Dévéhat [1,*,†], Solenn Ferron [1], Isabelle Rouaud [1], Pierre Le Pogam [1,2], Laurence Cornevin [1], Michel Bertrand [3] and Joël Boustie [1]

[1] ISCR –UMR CNRS 6226; Faculté des Sciences Pharmaceutiques et Biologiques, Institut des Sciences Chimiques de Rennes, Université Rennes 1, 2 Av. du Pr. Léon Bernard, 35043 Rennes Cedex, France; solenn.ferron@univ-rennes1.fr (S.F.); isabelle.rouaud@univ-rennes1.fr (I.R.); pierre.lepogam.alluard@gmail.com (P.L.P.); laurence.cornevin@univ-rennes1.fr (L.C.); joel.boustie@univ-rennes1.fr (J.B.)

[2] IETR-UMR CNRS 6164, Institut d'Électronique et de Télécommunications de Rennes, Université Rennes 1, 263 Av. du Général Leclerc, 35042 Rennes Cedex, France.

[3] French Lichenological Society- Station d'écologie forestière- Route de la tour Dénecourt, 77300 Fontainebleau/Avon, France; bertrand.mic@wanadoo.fr

* Correspondence: beatrice.legouin@univ-rennes1.fr (B.L.); francoise.le-devehat@univ-rennes1.fr (F.L.-L.D.); Tel.: +33-223234809 (B.L.); +33-223234816 (F.L.-L.D.).

† These authors contributed equally to this paper

1 Mass spectrometry data 3

2 NMR data 3

3 Commercial organic sunscreens 8

4 New method for photoprotective indexes' determination 9

5 Photostability .... **Error! Bookmark not defined.**

6 Photoprotective indexes in synergy's study 14

7 Antioxidant results in synergy studies 15

8 Photocytotoxicity results in synergy studies 16

References 18

Captions

Spectrum S1 ^{1}H NMR spectrum in $CDCl_3$ + d_6-acetone of the extract of *Vulpicida pinastri*

Spectrum S2a ^{1}H NMR spectrum in $CDCl_3$ of vulpinic acid (**1**)

Spectrum S2b ^{13} C NMR spectrum in $CDCl_3$ of vulpinic acid (**1**)

Spectrum S3a ^{1}H NMR spectrum in $CDCl_3$ of pinastric acid (**2**)

Spectrum S3b ^{13}C NMR spectrum in d_6-acetone of pinastric acid (**2**)

Spectrum S4 ^{1}H NMR spectrum in $CDCl_3$ of the extract of (+)-usnic acid (**3**)

Figure S1. Comparison of SPF values of authorized UV filters determined using PMMA plates and from experimental data in solution. (Correlation diagram presented in window).

Figure S2. PF-UVA and critical wavelength values of organic UV filters.

Figure S3. SUI and ISP values of organic UV filters.

Figure S4. UV spectra of OMC and lichen compounds before (in blue) and after UVA (red) and UVB (green) irradiation

Figure S5. Calculated and experimental photoprotective indexes of the pinastric and usnic acids mixture.

Figure S6. Experimental photoprotection indexes of the pinastric and usnic acids mixture (2+3) before and after UV irradiation

Figure S7. Isobologram analysis to evaluate the combination effect of vulpinic acid and usnic acid on superoxide scavenging activity (evaluated via NBT assay). Combination Index (CI) values at 0%, 25%, 50%, 75%, 100% of IC_{50} indicated the efficacy of both compounds at different ratio : CI < 1 means synergy, CI = 1 means additivity, CI > 1 means antagonism.

Figure S8. Cytotoxicity before and after irradiation under UVA of the combination effect of pinastric acid and usnic acid on HaCaT cells in the range of concentrations to observe a synergistic effect for antioxidant activity.

Table S1. Results of exact mass measurements performed from the mass spectrum of the Figure. 3 related to the PI-DART-MS of a whole piece of *Vulpicida pinastri*.

Table S2. ^{1}H and ^{13}C NMR spectroscopic data for compounds 1 and 2 ($CDCl_3$ and/or d_6-acetone, 300 MHz for ^{1}H NMR and 75 MHz for ^{13}C NMR)

Table S3. Characteristics of the commercial organic sunscreens

Table S4. Photoprotection indexes of organic filters from experimental data obtained in a 96-wells plate (grey line) *vs* data obtained in a cuvette (white line).

Table S5. Superoxide anion scavenging activity (% inhibition) when compound 1 and 3 are combinated

Table S6. Superoxide anion scavenging activity (% inhibition) when compound 2 and 3 are combinated

Table S7. Percentage of cytotoxicity of compound 1 and 3 before and after UVA

Table S1. Percentage of cytotoxicity of compound **2** and **3** before and after UVA irradiation

Mass spectrometry data

Table S2. Results of exact mass measurements performed from the mass spectrum of the Figure 1 related to the PI-DART-MS of a whole piece of *Vulpicida pinastri*. Note that the detected species arose as singly charged species

Measured Mass	Proposed Formulae	Calculated Mass (error in ppm)
263.07160	$C_{17}H_{11}O_3$	263.07027 (5.05)
291.06537	$C_{18}H_{11}O_4$	291.06519 (0.62)
319.11737	$C_{17}H_{19}O_6$	319.11761 (−2.47)
321.07627	$C_{19}H_{13}O_5$	321.07575 (1.62)
323.09142	$C_{19}H_{15}O_5$	323.09140 (0.06)
345.09688	$C_{18}H_{17}O_7$	345.09688 (0.00)
353.10130	$C_{20}H_{17}O_6$	353.10196 (−1.87)

NMR data

Spectrum S1. ^1H NMR spectrum in $CDCl_3$ + *d_6*-acetone of the extract of *Vulpicida pinastri.*

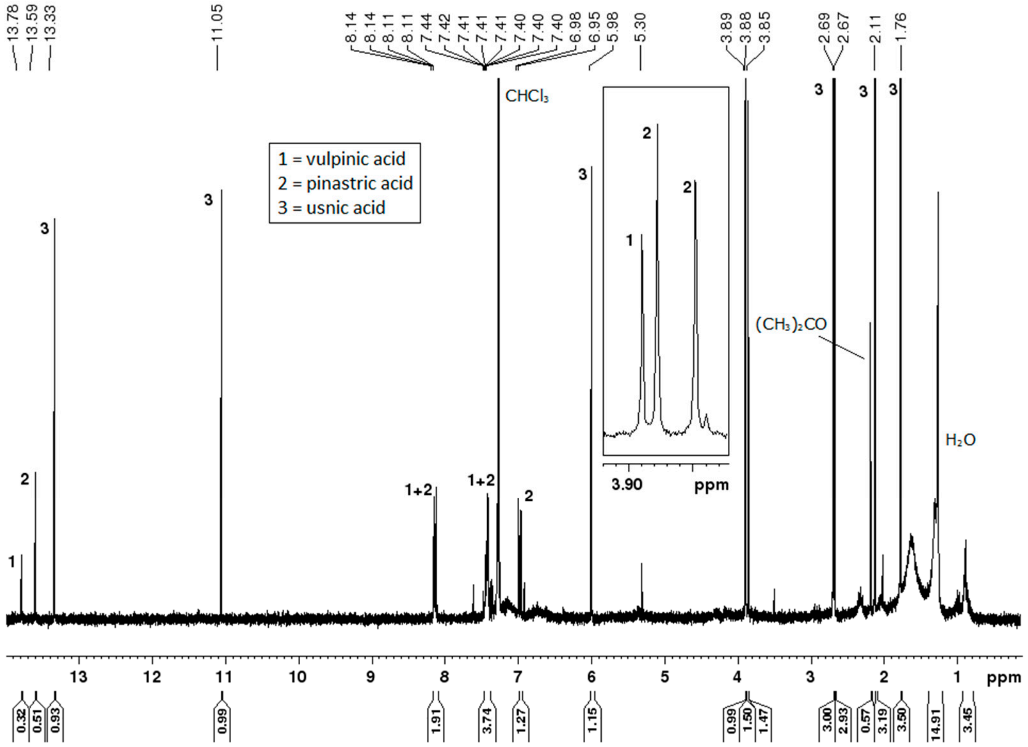

Spectrum S2a. ^{1}H NMR spectrum in $CDCl_3$ of vulpinic acid (**1**).

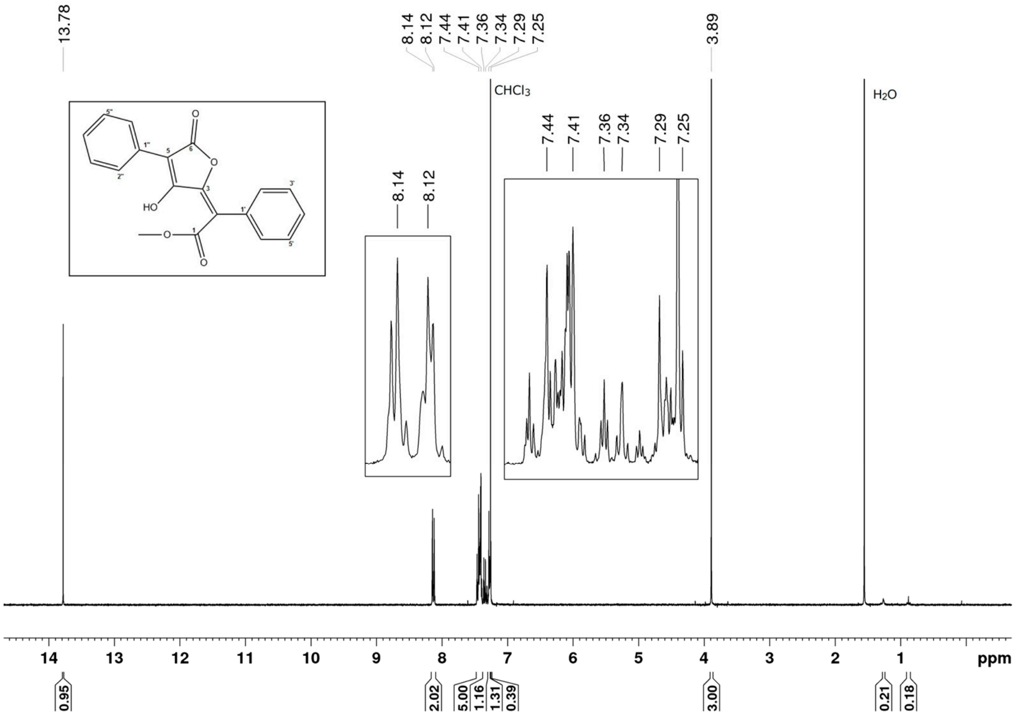

Spectrum S2b. [13] C NMR spectrum in CDCl3 of vulpinic acid (**1**).

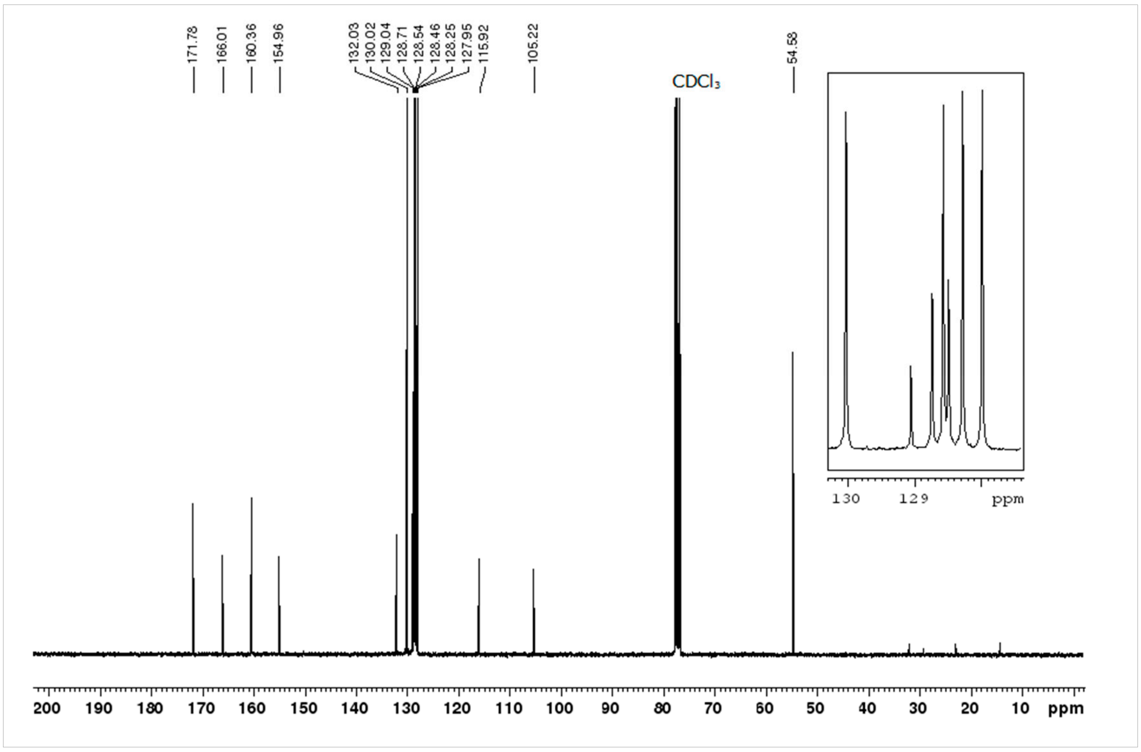

Spectrum S3a. ^{1}H NMR spectrum in $CDCl_3$ of pinastric acid (**2**).

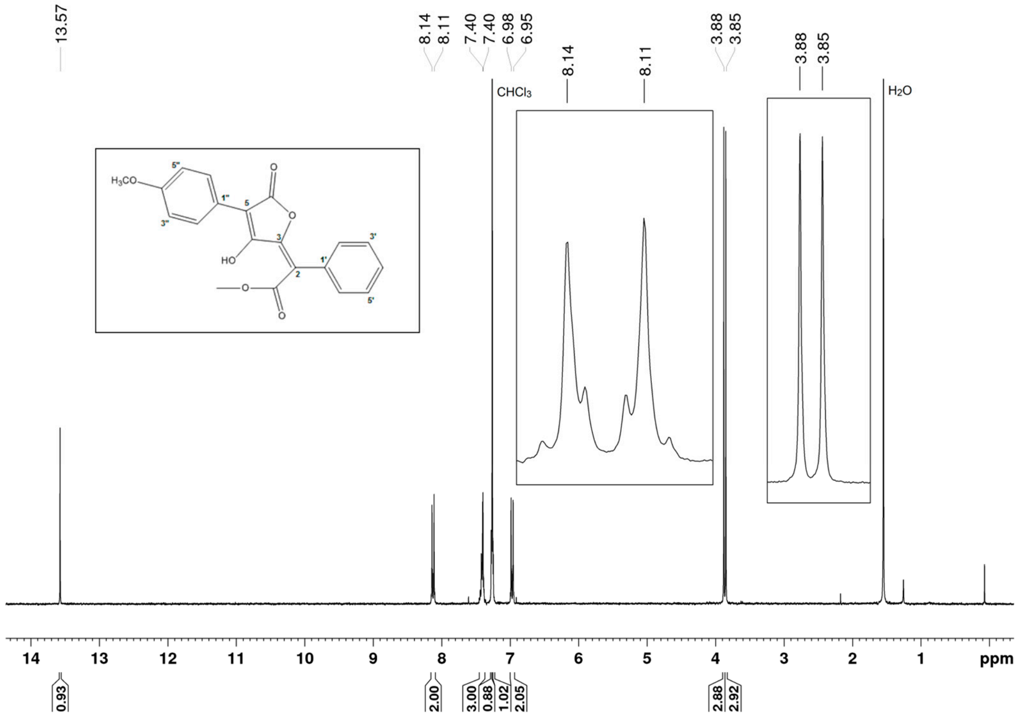

Spectrum S3b. ^{13}C NMR spectrum in d_6-acetone of pinastric acid (**2**).

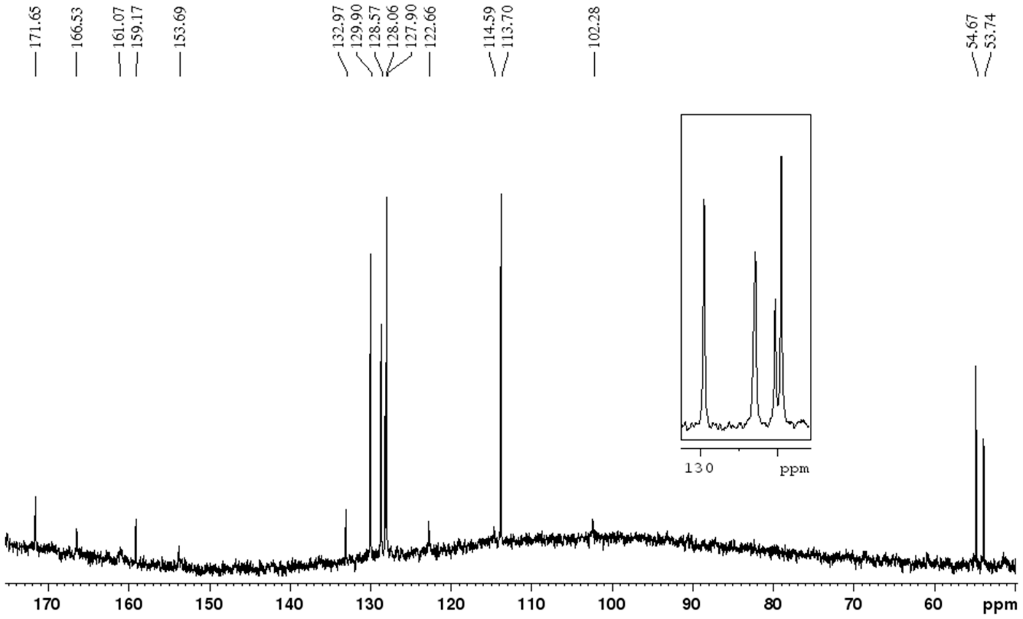

Spectrum S4. ^{1}H NMR spectrum in $CDCl_3$ of usnic acid (**3**).

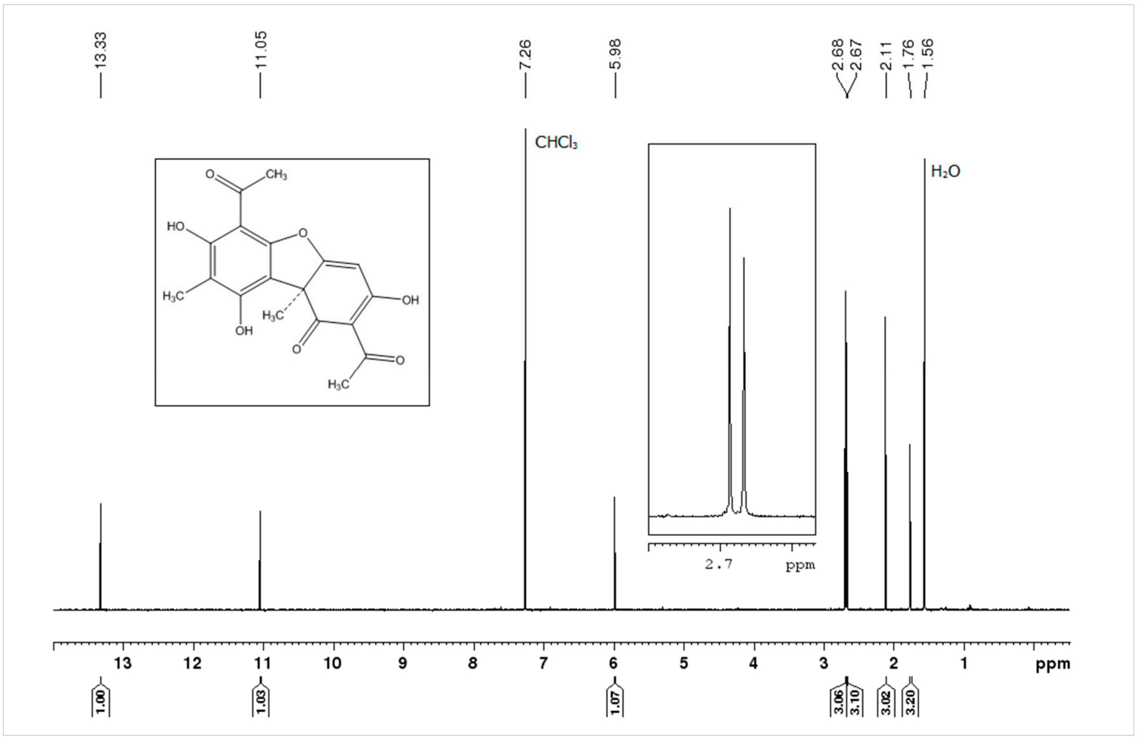

Table S3. ^{1}H and ^{13}C NMR spectroscopic data for compounds **1** and **2** ($CDCl_3$ and/or d_6-acetone, 300 MHz for ^{1}H NMR and 75 MHz for ^{13}C NMR)

	**Vulpinic Acid**		**Pinastric Acid**	
Position	δ_C (ppm)	δ_H (ppm) (mult., *J* in Hz)	δ_C (ppm)	δ_H (ppm) (mult., *J* in Hz)
1	129	-	122.7	-
2	127.9	8.13 (m, 1H)	127.9	8.1 (d, 9.1, 1H)
3	128.5	7.43 (m, 2H)	113.7	6.97 (d, 9.1, 1H)
4	128.5	7.4 (m, 1 H)	161	-
5	128.5	7.43 (m, 1H)	113.7	6.97 (d, 9.1, 1H)
6	127.9	8.13 (m, 1H)	127.9	8.1 (d, 9.1, 1H)
1′	132.0	-	133	-
2′	130.0	7.30 (m, 1H)	129.9	7.25 (m, 1H)
3′	128.3	7.43 (m, 1H)	128.6	7.4 (m, 1H)
4′	128.7	7.43 (m, 1H)	128.06	7.4 (m, 1H)
5′	128.3	7.43 (m, 1H)	128.6	7.4 (m, 1H)
6′	130.0	7.30 (m, 1H)	129.9	7.25 (m, 1H)
7′	115.9	-	114.6	-
8′	155.0	-	153.7	-
9′	160.3	-	159.2	-
10′	105.2	-	102.3	-
11′	166.	-	166.5	-
12′	171.8	-	171.8	-
OCH_3-4	-	-	53.7	3.85 (s, 3H)
OCH_3-12′	54.6	3.89 (s, 3H)	54.7	3.88 (s, 3H)
OH-9′	-	13.78 (s, 1H	-	13.57 (s, 1H)

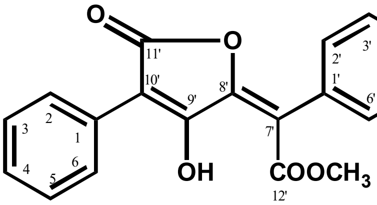

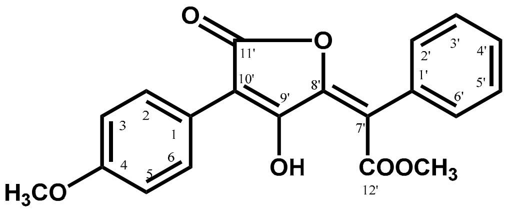

Commercial organic sunscreens

Table S4. Characteristics of the commercial organic sunscreens.

INCI Name	Supplier	Maximum Authorized Concentration (%)	UV Range
4-Methylbenzylidene camphor (4-MBC)	Merck	4	UVB
Bis-Ethylhexyloxyphenol Methoxytphenyl Triazine (Anisotriazine)	Ciba	10	UVA+UVB
Buthyl Methoxydibenzoylmethane (Avobenzone)	Merck	10	UVA
Diethylhexyl butamido triazone (Uvasorb HEB)	Créations couleur	10	UVB
Homosalate	Merck	10	UVB
Octocrylene	BASF	10	UVA+UVB
Ethylhexyl dimethyl PABA (ODM-PABA)	Merck	8	UVB
Ethylhexyl methoxycinnamate (OMC)	Merck	10	UVB
Ethylhexyl triazone (Octyltriazone)	BASF	5	UVB
Benzophenone-3 (Oxybenzone)	BASF	10	UVA+UVB
PEG-25 PABA	BASF	10	UVB
Phenylbenzimidazole sulfonic acid (PB SULF)	Merck	8	UVB
Polysilicone 15 (PS15)	Roche	10	UVB

Merck (Fontenay sous Bois, France) – BASF (Levallois-Perret, France) – Créations couleur (Dreux, France) – Roche (Fontenay sous Bois, France) – Ciba (Grenzach-Wyhlen, Germany)

New method for Photoprotective Indexes' Determination

A new method for photoprotective indexes' determination was developed to face the low availability of products such as secondary metabolites stemming from natural products. The method, first developed with commercial filters, allows the determination of SPF, PF-UVA, λ_C, SUI and ISP indexes.

Emulsion Preparation

An emulsion O/W was first prepared by dissolving 10 g of sodium lauryl sulfate in 20 g distilled water. Then, under a vigorous mix in a blender, 10 g of liquid paraffin was incorporated to obtain a homogeneous emulsion. The quantity of emulsion required to perform the analysis is much less important than the quantity prepared due to device size. The efficiency of the emulsion was then investigated after freezing of aliquots at −4 °C. The emulsion is stable during 3 months.

In Vitro Sun Protection Indexes Determination

The experimental data were recorded from ethanolic solutions prepared according the expected compound percentage. Absorbances (A_λ) were then transferred for calculations into a specially dedicated Excel spreadsheet. Transmittance (T_λ) was calculated from absorbance according to equation 1. The experimental values have directly resulted in both SPF and PF-UVA values [1] (equations 2 and 3), critical wavelength (λ_c) [1] (equation 4), Spectral Uniformity Index (SUI) [2] (equation 5), Ideal Spectral Profile (ISP) [3](equation 6).

$$A(\lambda)=10^{-T} \quad (1)$$

$$\mathrm{UV-PF} = \frac{\sum_{290}^{400} E_\lambda I_\lambda \Delta_\lambda}{\sum_{290}^{400} E_\lambda I_\lambda T_\lambda \Delta_\lambda} \quad (2)$$

$$\mathrm{UVA-PF} = \frac{\sum_{320}^{400} E_\lambda I_\lambda \Delta_\lambda}{\sum_{320}^{400} E_\lambda I_\lambda T_\lambda \Delta_\lambda} \quad (3)$$

E_λ is the spectral irradiation of terrestrial sunlight at λ, I_λ is the erythemal action spectrum at λ and T_λ is the spectral transmittance of the sample at λ. SPF values are means of different values calculated from ($E_\lambda \times I_\lambda$)relative to Mexico, Melbourne, [1,4,5].

$$\lambda_C = 0.9\int_{290}^{400} A(\lambda)d\lambda \quad (4)$$

$$SUI = \frac{\sum_{290}^{380} A_{\lambda}}{\sum_{290}^{380} \left| A_{\lambda} - \overline{A} \right|} \tag{5}$$

$$ISP\ (\%) = \frac{\sum_{290}^{400} \left| A_{\lambda} - \hat{A}_{\lambda} \right|}{\sum_{290}^{400} A_{\lambda}} \tag{6}$$

where A_λ is the spectral absorbance at λ and $\hat{A}_\lambda$ is the ideal spectral absorbance at λ. $\hat{A}_\lambda$ is equal to the mean absorbance between 290 and 385 nm for all wavelengths in this spectral interval. Between 385 and 400 nm, $\hat{A}_\lambda$ is given as:

$$\hat{A}_{\lambda} = \hat{A}_{385}\ x\ \frac{400 - \lambda}{15} \tag{7}$$

$$r = \sqrt{1 - SSE/SST} \tag{8}$$

where SST is the sum of squared deviation from the mean absorbance over the spectral band 290–400 nm and SSE is the sum of the squared deviations from the ideal absorbance at each wavelength [3]

$$SST = \sum_{290}^{400} \left| A_{\lambda} - \overline{A} \right|^{2} \tag{9}$$

$$SSE = \sum_{290}^{400} \left| A_{\lambda} - \hat{A}_{\lambda} \right|^{2} \tag{10}$$

Method's Development in Cuvette

Twelve commercial UV-filters spanning the 1 to 25 SPF range (Table S3) were used to develop the protocol. Experimental SPF values were compared with published data which were considered as reference values in this work [6–8] (Figure S1). For each compound, all indexes were determined from equations described above. The solubility has important bearing on SPF values drawn from assays conducted on anizotriazine and OMC. First, anisotriazine exhibited a SPF value of 22.8 in THF, close to the published value (SPF = 23.9), while the SPF value was 1.8 in DMSO where it was slightly soluble. Compounds were then dissolved either in THF or in DMSO depending on their solubility. Conversely, results were not statistically different for assays carried out on OMC, dissolved either in THF or in DMSO where it was soluble, SPF = 9.6 in THF and SPF = 10.1 in DMSO.

The SPF values were within the span 3.6 to 23.9 and were well correlated with the reference values ($r^2 = 0.977$). As a general trend, this new method well answered in an extended range of values though it underestimated them, suggesting even higher photoprotection when investigated in formulation.

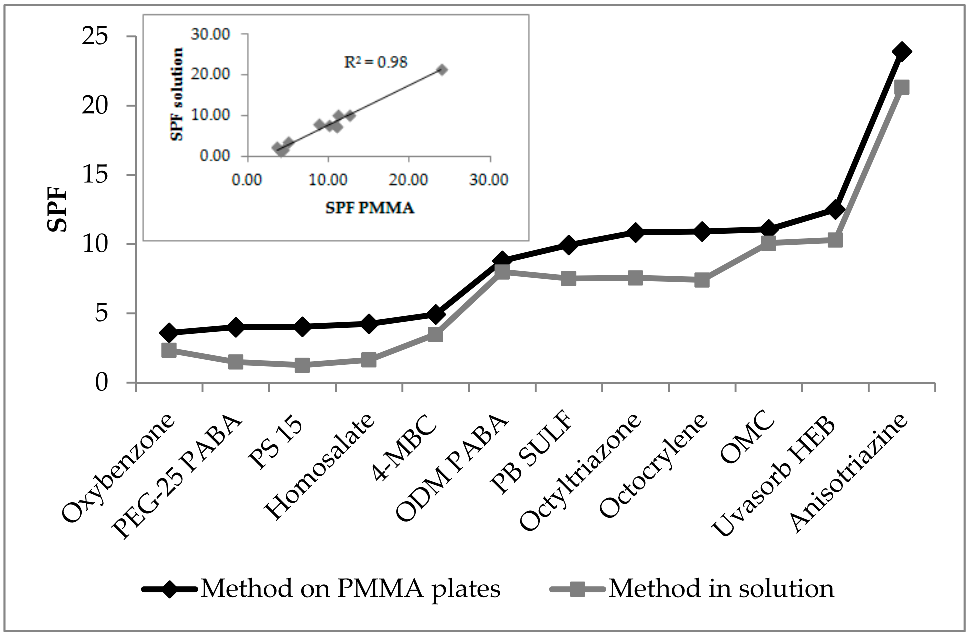

Figure S1. Comparison of SPF values of authorized UV filters determined using PMMA plates and from experimental data in solution. (Correlation diagram presented in window).

All the results of critical wavelengths and PF-UVA are summarized in Figure S2.

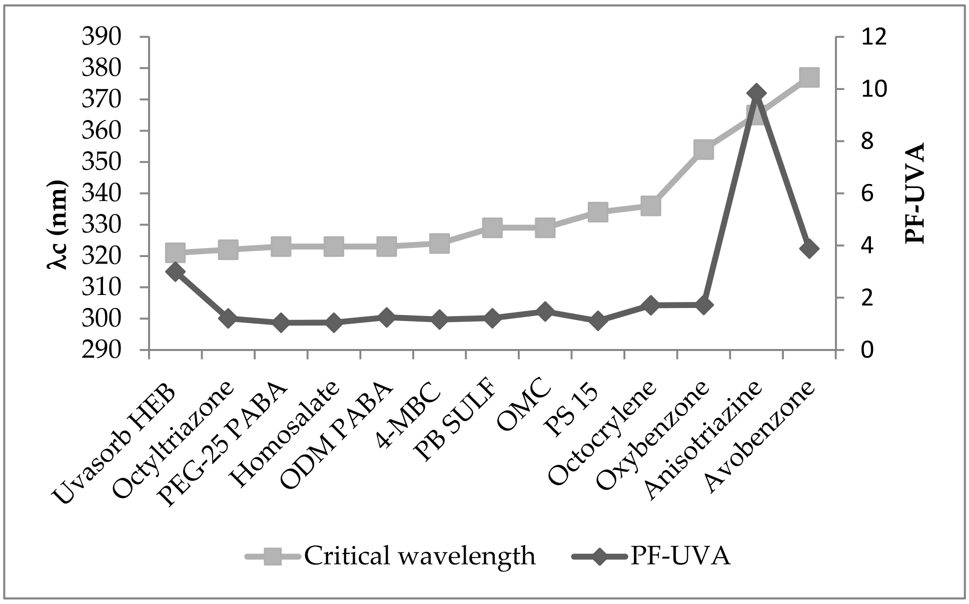

Figure S2. PF-UVA and critical wavelength values of organic UV filters.

All the λc values were below 370 nm and PF-UVA values were under 3 except for anisotriazine (Figure S2). This was in agreement with the classification of these filters in the UVB and UVA+UVB range (Table S3). The ability of the method to discriminate a UVA filter *via* these indexes, was illustrated with avobenzone. Its critical wavelength and its PF-UVA value, respectively 377 nm and 3.9, were consistent with the restricted UVA filter profile of this compound.

Recently, new indexes of broad spectrum protection (SUI and ISP) were reported by Diffey [2,3] to focus on protection across the entire ultraviolet spectrum. They calculated the goodness of fit of the spectral profile to the ideal flat profile. The higher the SUI indexes, the better the product meets the ideal spectral requirement of a uniform absorption sunscreen throughout the ultraviolet spectrum. So does a weak ISP value. Anisotriazine, avobenzone, oxybenzone and octocrylene differed clearly

with their index values in good agreement with their well known protection in the UVA range whereas the others are strict UVB filters (Figure S3).

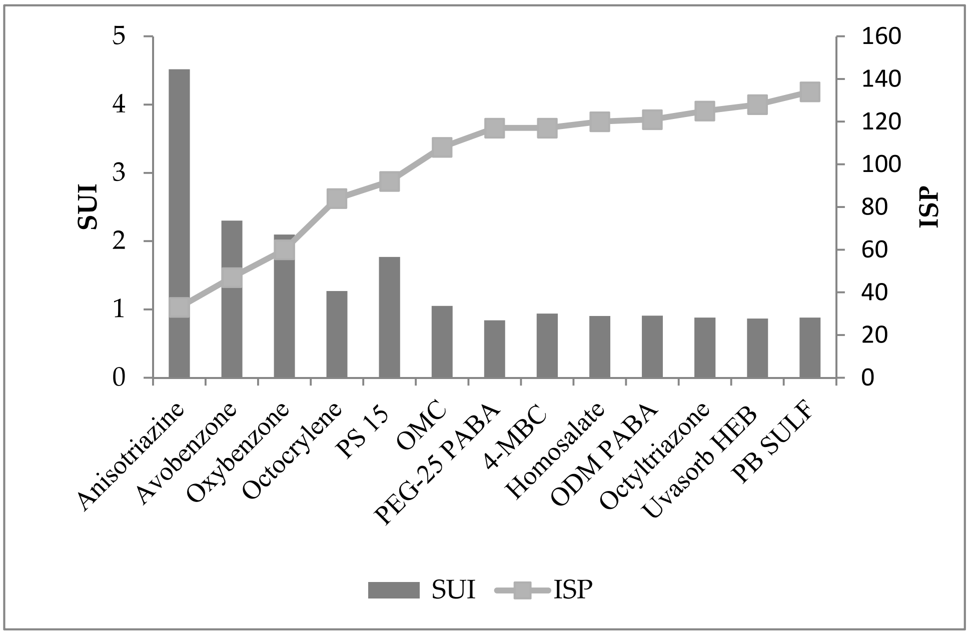

Figure S3. SUI and ISP values of organic UV filters.

Arising from these results on twelve commercial organic filters, all indexes are in agreement with their well-known photoprotective properties. This new method allows the identification of UVA, UVB or (UVA + UVB) filter candidates using absolute and relative indexes of photoprotection (SPF, PF-UVA, λc, SUI, ISP). .

96-well Plates Indexes vs Cuvette Indexes

In a second time, in order to use the method for high throughput screening, we have adapted it for 96-well plate. 180 μL of solution S4 fill the well, absorbances were recorded and results were obtained as described above. Four commercial UV filters (OMC, anisotriazine, Uvasorb HEB, oxybenzone) were selected to cover a large SPF range. The adequacy of the results was evaluated by comparing the photoprotective indexes of these four filters obtained from absorbances recorded in cuvette and in a 96-well plates. All results collected in Table S5 were self-consistent and have validated the high throughput screening method.

Table S5. Photoprotection indexes of organic filters from experimental data obtained in a 96-wells plate (grey line) *vs* data obtained in a cuvette (white line). CV in % in brackets.

	**OMC**	**Anisotriazine**	**Uvasorb**	**Oxybenzone**
SPF	10.4	22.3	9.0	3.4
	10.1 (−2.9)	22.8(2.2)	10.3(14.4)	2.3(−26.5)
PF-UVA	1.5	10.2	1.26	1.9
	1.5 (0)	9.8(3.9)	3(138.1)	1.7(−10.5)
λc (nm)	330	366	322	349
	329 (−0.3)	365(−0.3)	321(−0.3)	354(1.4)
ISP	105	71	124	64
	107 (1.9)	70(−1.4)	128(3.22)	60(−6.25)
SUI	1.1	4.9	0.9	1.9
	0.9(−18.1)	4.5(−8.2)	0.9(0)	2.1(10.5)

Photostability

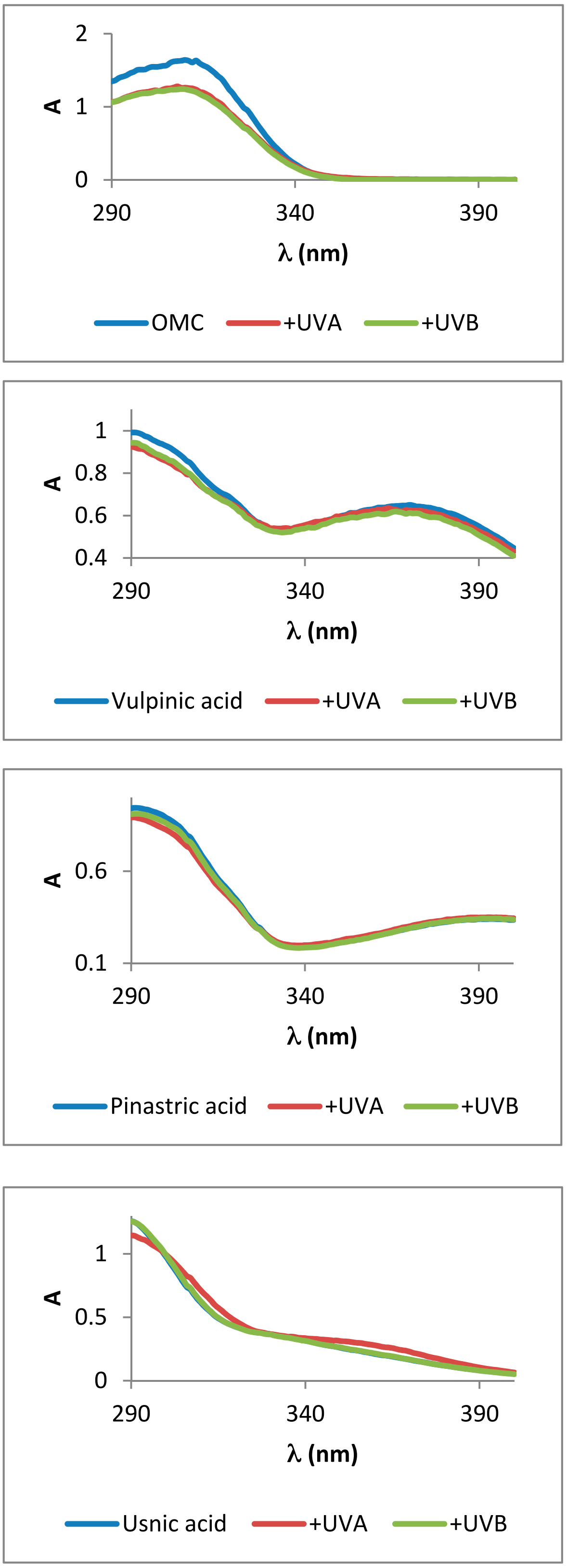

Figure S4. UV spectra of OMC and lichen compounds before (blue) and after UVA (red) and UVB (green) irradiation

Photoprotective Indexes in Synergy's Study

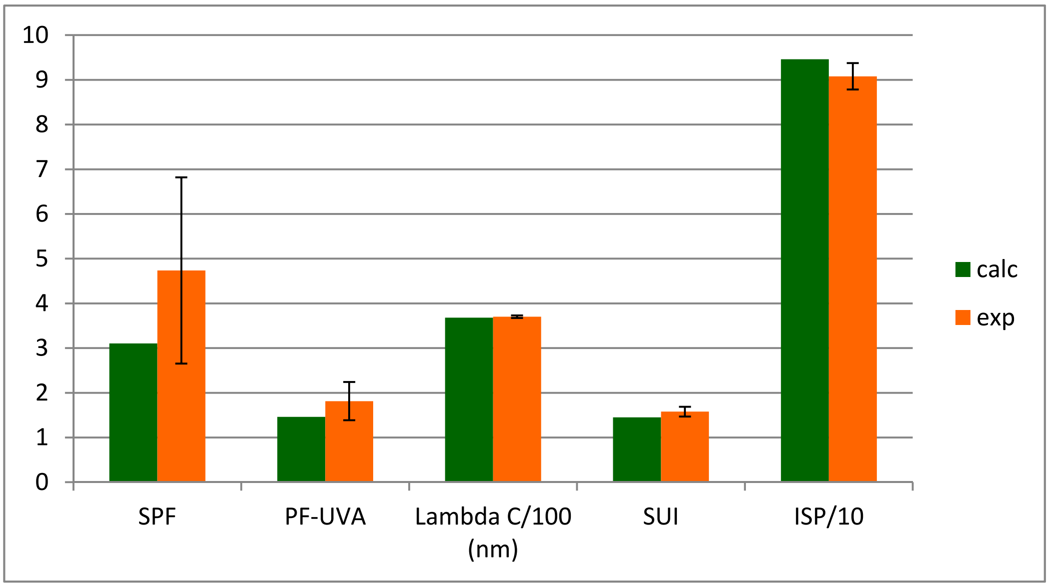

Figure S5. Calculated (calc) and experimental (exp) photoprotective indexes of the pinastric and usnic acids mixture.

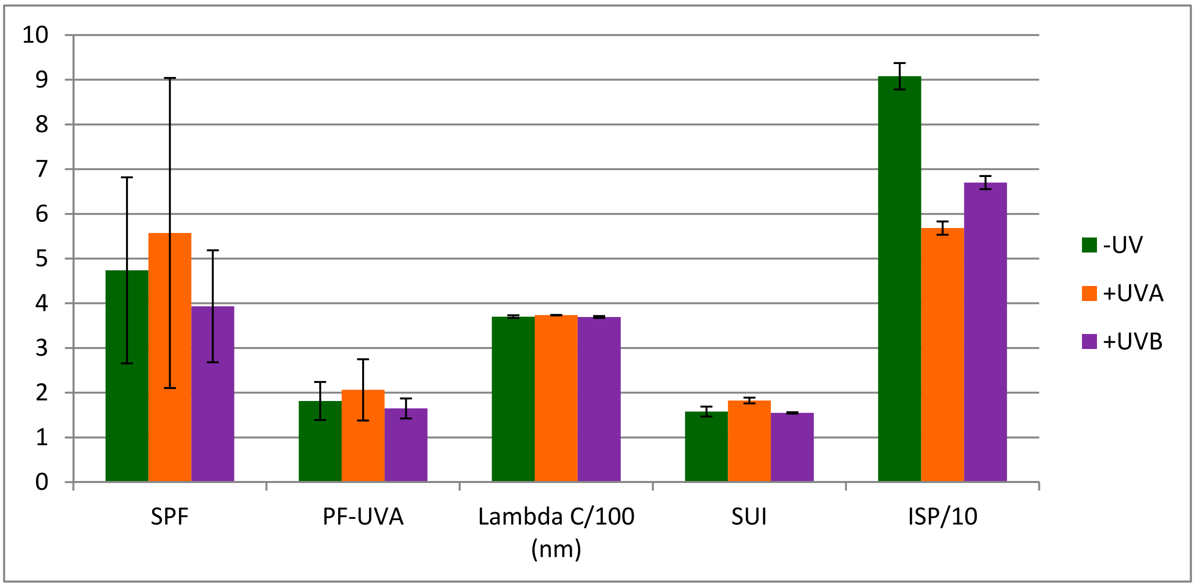

Figure S6. Experimental photoprotection indexes of the pinastric and usnic acids mixture (**2**+**3**) before and after UV irradiation.

Antioxidant Results in Synergy Studies

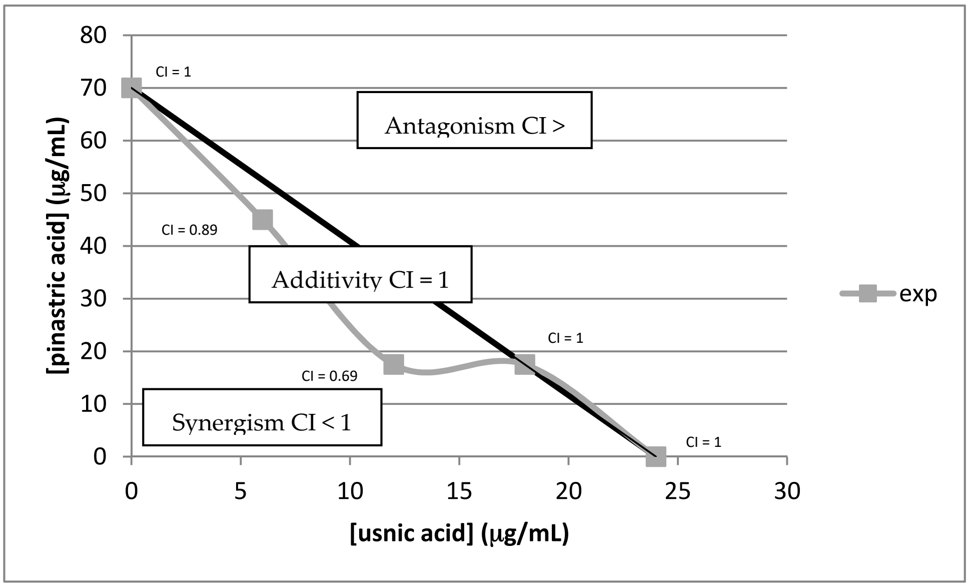

Figure S7. Isobologram analysis to evaluate the combination effect of vulpinic acid and usnic acid on superoxide scavenging activity (evaluated via NBT assay). Combination Index (CI) values at 0%, 25%, 50%, 75%, 100% of IC_{50} indicated the efficacy of both compounds at different ratio.

Table S6. Superoxide anion scavenging activity (% inhibition) when compound **1** and **3** are combinated.

			Usnic Acid (μg/mL)				
			**0% IC** $_{50}$ (0 μg/mL)	**25% IC** $_{50}$ (6.0 μg/mL)	**50% IC** $_{50}$ (12.0 μg/mL)	**75% IC** $_{50}$ (18.0 μg/mL)	**100% IC** $_{50}$ (24.0 μg/mL)
vulpinic acid (μg/mL)	**0% IC** $_{50}$	(0 μg/mL)	0.0	32.0	40.0	46.0	50.0
	**25% IC** $_{50}$	(7.5 μg/mL)	22.0	38.9	45.3	49.3	52.6
	**50% IC** $_{50}$	(15.0 μg/mL)	30.0	37.9	46.7	55.4	60.5
	**75% IC** $_{50}$	(22.5 μg/mL)	42.0	52.3	56.6	64.1	68.8
	**100% IC** $_{50}$	(30.0 μg/mL)	50.0	53.2	63.5	70.2	71.0

Table S7. Superoxide anion scavenging activity (% inhibition) when compound **2** and **3** are combinated.

			Usnic Acid (μg/mL)				
			**0% CI** $_{50}$ (0 μg/mL)	**25% CI** $_{50}$ (6.0 μg/mL)	**50% CI** $_{50}$ (12.0 μg/mL)	**75% CI** $_{50}$ (18.0 μg/mL)	**100% CI** $_{50}$ (24.0 μg/mL)
pinastric acid (μg/mL)	**0% CI** $_{50}$	(0 μg/mL)	0.0	32.0	40.0	46.0	50.0
	**25% CI** $_{50}$	(17.5 μg/mL)	25.0	42.9	50.0	51.7	55.3
	**50% CI** $_{50}$	(35.0 μg/mL)	37.0	45.3	50.2	57.9	67.2
	**75% CI** $_{50}$	(52.5 μg/mL)	44.0	55.1	64.1	71.7	74.3
	**100% CI** $_{50}$	(70.0 μg/mL)	50.0	57.7	65.4	73.0	78.6

Photocytotoxicity Results in Synergy Studies

Table S8. Percentage of cytotoxicity of compound **1** and **3** before and after UVA.

	**Usnic acid in µg/mL**					
	Without UVA	**0**	**5**	**20**	**50**	**100**
Vulpinic acid (µg/mL)	**0**	0.0	3.1	4.9	93.3	95.1
	**5**	0.0	0.0	3.1	40.8	90.8
	**20**	0.0	0.0	3.3	63.2	91.9
	**50**	0.0	0.0	1.6	52.8	92.4
	**100**	0.0	0.0	12.8	66.3	98.1
	**Usnic acid in µg/mL**					
	With UVA	**0**	**5**	**20**	**50**	**100**
Vulpinic acid (µg/mL)	**0**	0.0	19.3	26.4	52.0	95.3
	**5**	0.0	15.7	8.5	66.6	76.4
	**20**	0.0	11.5	7.4	63.0	77.2
	**50**	10.5	7.0	8.5	57.1	71.4
	**100**	20.5	6.0	2.3	69.6	86.3

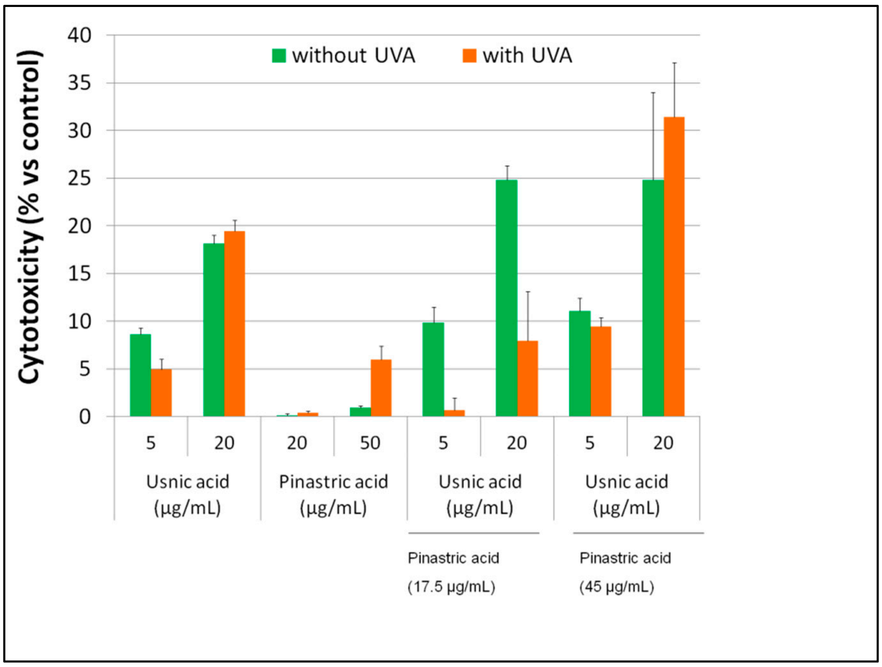

Figure S8. Cytotoxicity before and after irradiation under UVA of the combination effect of pinastric acid and usnic acid on HaCaT cells in the range of concentrations to observe a synergistic effect for antioxidant activity.

Table S9. Percentage of cytotoxicity of compound **2** and **3** before and after UVA irradiation.

		**Usnic Acid in μg/ml**				
	Without UVA	**0**	**5**	**20**	**50**	**100**
pinastric acid (μg/mL)	**0**	0.0	8.6	18.1	57.5	95.1
	**5**	1.3	19.6	36.2	60.6	94.2
	**20**	0.1	9.9	24.8	49.2	88.3
	**50**	0.9	11.1	26.8	50.6	93.7
	**100**	0.0	12.3	31.3	66.8	95.8

		**Usnic Acid in μg/ml**				
	With UVA	**0**	**5**	**20**	**50**	**100**
pinastric acid (μg/mL)	**0**	0.0	4.9	19.4	74.2	95.1
	**5**	0.0	12.6	29.4	50.7	83.0
	**20**	0.4	0.7	7.9	59.4	83.3
	**50**	5.9	9.	31.4	56.7	87.2
	**100**	40.4	21.3	50.4	69.5	88.4

References

1. Comité de Liaison des Industries de la Parfumerie (Colipa), Colipa guidelines 2007. In vitro method for determination of the UVA protection factor and critical wavelength values of sunscreen products, Brussels, Belgium, 2007.
2. Diffey, B. Spectral uniformity: a new index of broad spectrum (UVA) protection. *Int. J. Cosmet. Sci.* **2009**, *31*, 63–68.
3. Diffey, B.L.; Brown, M.W. The ideal spectral profile of topical sunscreens. *Photochem. Photobiol.* **2012**, *88*, 744–747.
4. Comité de Liaison des Industries de la Parfumerie, (Colipa), Colipa guidelines 2011 In vitro Method for the determination of the UVA protection factor and "critical wavelength" values of sunscreen products.,Brussels, Belgium, 2011.
5. Diffey, B.L.; Robson, J. A new substrate to measure sunscreen protection factors throughout the ultraviolet spectrum. *J Soc Cosmet Chem.* **1989**, *40*, 127–133.
6. Couteau, C.; Pommier, M.; Paparis, E.; Coiffard, L.J.M. Study of the efficacy of 18 sun filters authorized in European Union tested in vitro. *Pharmazie* **2007**, *62*, 449–452.
7. Couteau, C.; Faure, A.; Fortin, J.; Paparis, E.; Coiffard, L.J.M. Study of the photostability of 18 sunscreens in creams by measuring the SPF in vitro. *J. Pharm. Biomed. Anal.* **2007**, *44*, 270–273.
8. El-Boury, S.; Couteau, C.; Boulande, L.; Paparis, E.; Coiffard, L.J.M. Effect of the combination of organic and inorganic filters on the Sun Protection Factor (SPF) determined by in vitro method, *Int. J. Pharm.* **2007**, *340,* 1–5.
